# Development of a Plasmid Shuttle Vector System for Genetic Manipulation of Chlamydia psittaci

**DOI:** 10.1128/mSphere.00787-20

**Published:** 2020-08-26

**Authors:** Kensuke Shima, Mary M. Weber, Christiane Schnee, Konrad Sachse, Nadja Käding, Matthias Klinger, Jan Rupp

**Affiliations:** a Department of Infectious Diseases and Microbiology, University of Lübeck, Lübeck, Germany; b Department of Microbiology and Immunology, University of Iowa Carver College of Medicine, Iowa City, Iowa, USA; c Institute of Molecular Pathogenesis, Friedrich-Loeffler-lnstitut (Federal Research Institute for Animal Health), Jena, Germany; d RNA Bioinformatics and High-Throughput Analysis, Faculty of Mathematics and Computer Science, Friedrich-Schiller-Universität Jena, Jena, Germany; e Institute of Anatomy, University of Lübeck, Lübeck, Germany; f German Center for Infection Research (DZIF), partner site Hamburg-Lübeck-Borstel, Germany; University of Kentucky

**Keywords:** *Chlamydia psittaci*, Gram-negative bacteria, intracellular bacteria, plasmid shuttle vector, transformation

## Abstract

The obligate intracellular bacterium Chlamydia psittaci is a known avian pathogen causing psittacosis in birds and is capable of zoonotic transmission. In human pulmonary infections, C. psittaci can cause pneumonia associated with significant mortality if inadequately diagnosed and treated. Although intracellular C. psittaci manipulates host cell organelles for its replication and survival, it has been difficult to demonstrate host-pathogen interactions in C. psittaci infection due to the lack of easy-to-handle genetic manipulation tools. Here, we show the genetic transformation of C. psittaci using a plasmid shuttle vector that contains a controllable gene induction system. The 7,553-bp plasmid p01DC12 was prepared from the nonavian C. psittaci strain 01DC12. We constructed the shuttle vector pCps-Tet-mCherry using the full sequence of p01DC12 and the 4,449-bp fragment of Chlamydia trachomatis shuttle vector pBOMB4-Tet-mCherry. pCps-Tet-mCherry includes genes encoding the green fluorescent protein (GFP), mCherry, and ampicillin resistance (AmpR). Target genes can be inserted at a multiple cloning site (MCS). Importantly, these genes can be regulated by a tetracycline-inducible (tet) promoter. Using the pCps-Tet-mCherry plasmid shuttle vector, we show the expression of GFP, as well as the induction of mCherry expression, in C. psittaci strain 02DC15, which belongs to the avian C. psittaci 6BC clade. Furthermore, we demonstrated that pCps-Tet-mCherry was stably retained in C. psittaci transformants. Thus, our C. psittaci plasmid shuttle vector system represents a novel targeted approach that enables the elucidation of host-pathogen interactions.

**IMPORTANCE** Psittacosis, caused by avian C. psittaci, has a major economic impact in the poultry industry worldwide and represents a significant risk for zoonotic transmission to humans. In the past decade, the tools of genetic manipulation have been improved for chlamydial molecular studies. While several genetic tools have been mainly developed in Chlamydia trachomatis, a stable gene-inducible shuttle vector system has not to date been available for C. psittaci. In this study, we adapted a C. trachomatis plasmid shuttle vector system to C. psittaci. We constructed a C. psittaci plasmid backbone shuttle vector called pCps-Tet-mCherry. The construct expresses GFP in C. psittaci. Importantly, exogeneous genes can be inserted at an MCS and are regulated by a tet promoter. The application of the pCps-Tet-mCherry shuttle vector system enables a promising new approach to investigate unknown gene functions of this pathogen.

## Abstract (precis)

Psittacosis, caused by avian C. psittaci, has a major economic impact in the poultry industry worldwide and represents a significant risk for zoonotic transmission to humans. In the past decade, the tools of genetic manipulation have been improved for chlamydial molecular studies. While several genetic tools have been mainly developed in Chlamydia trachomatis, a stable gene-inducible shuttle vector system has not to date been available for C. psittaci. In this study, we adapted a C. trachomatis plasmid shuttle vector system to C. psittaci. We constructed a C. psittaci plasmid backbone shuttle vector called pCps-Tet-mCherry. The construct expresses GFP in C. psittaci. Importantly, exogeneous genes can be inserted at an MCS and are regulated by a tet promoter. The application of the pCps-Tet-mCherry shuttle vector system enables a promising new approach to investigate unknown gene functions of this pathogen.

## INTRODUCTION

The obligate intracellular Gram-negative bacterium Chlamydia psittaci (C. psittaci) is an important zoonotic pathogen that can be encountered in more than 400 bird species, as well as in sheep, cattle, swine, horses, goats, and cats (1–3). When avian C. psittaci strains infect humans, they can cause severe atypical pneumonia, with even a fatal outcome in some cases. In contrast, nonavian C. psittaci strains might be excluded as potential zoonotic risk factors since the case reports of human psittacosis usually include an avian source (1–3).

In host cells, chlamydiae undergo a unique developmental cycle that alternates between two distinct bacterial forms, infectious elementary bodies (EBs) and the replicating reticulate bodies (RBs). The developmental cycle is completed within the confines of a membrane-bound vacuole termed the inclusion (4). During infection, chlamydiae interact with various host cell organelles to acquire host-derived nutrients for their survival (5–7).

Genome sequence analysis of C. psittaci revealed a circular 1.2-Mb chromosome and an approximately 7.5-kb plasmid harboring seven to eight putative coding DNA sequences (CDS) (1, 8–10).

The lack of tools for genetic manipulation of chlamydiae hampered molecular research progress for many years. However, Binet and Maurelli first demonstrated transformation of C. psittaci, showing successful allelic exchange on the bacterial chromosome (11). Afterward, Wang et al. demonstrated a stable targeted genetic modification method of C. trachomatis using a plasmid shuttle vector (12). Their system has been modified for other *Chlamydia* species (spp.) including C. pneumoniae and C. muridarum, but not C. psittaci (12–14). To replicate efficiently, the plasmid shuttle vector usually has to match the chlamydial plasmid with the target host species due to the presence of replications barriers (13–15). In this study, we established a stable targeted genetic modification system for C. psittaci infections.

## RESULTS

### Construction of a plasmid shuttle vector for transformation of C. psittaci.

Plasmid DNA sequence comparison of a nonavian isolate C. psittaci 01DC12 (GenBank: HF545615.1) shares 99.97% identity to the avian isolate C. psittaci 6BC (GenBank: CP002587.1) (Fig. S1 in the supplemental material). In the amino acid sequence of protein coding regions, only one amino acid is different between pCps6BC and p01DC12 (Fig. S1). Due to the sequence similarity and biosafety considerations, the p01DC12 plasmid derived from C. psittaci 01DC12 was selected for construction of the shuttle vector.

10.1128/mSphere.00787-20.1 FIG S1 Plasmid sequence comparison of p01DC12 from C. psittaci 01DC12 and pCps6BC from C. psittaci 6BC. Only one amino acid is different between p01DC12 and pCps6BC. Download FIG S1, TIF file, 0.3 MB. Copyright © 2020 Shima et al. 2020 Shima et al. This content is distributed under the terms of the Creative Commons Attribution 4.0 International license.

**FIG 1:**
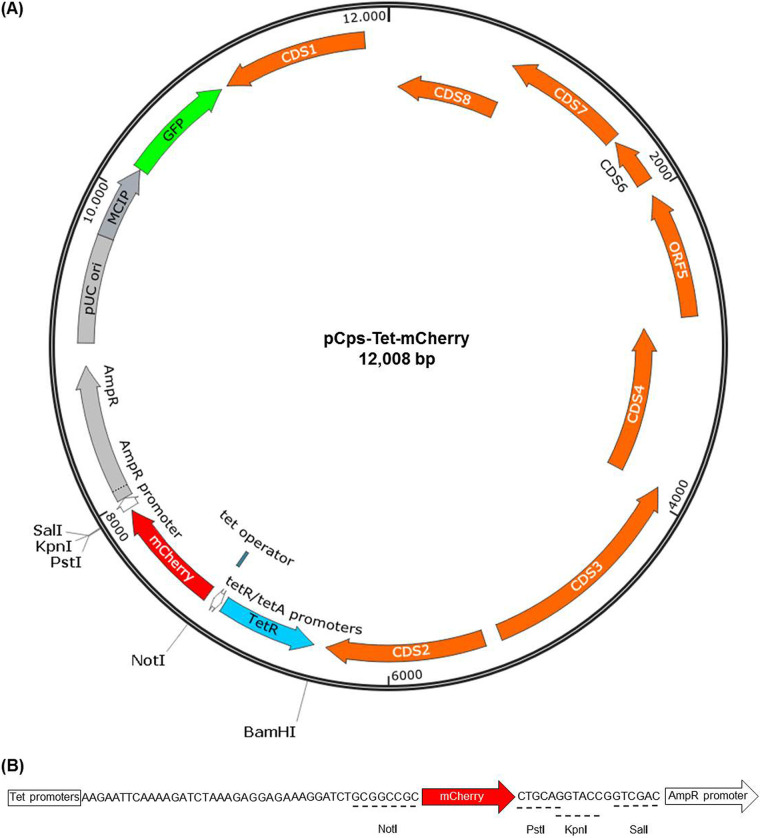
Map of the C. psittaci*-*derived shuttle vector pCps-Tet-mCherry. (A) The CDSs of p01DC12 are shown in orange. GFP, mCherry, and the Tet repressor are shown in green, red, and light blue, respectively. AmpR, pUC ori, and MCIP are shown in light gray. The full sequence of p01DC12 was amplified from C. psittaci 01DC12. Another fragment was amplified from pBOMB4-tet-mCherry. mCherry can be induced by a tetracycline-inducible promoter. (B) An MCS containing NotI, PstI, KpnI, and SalI restriction sites in pCps-Tet-mCherry.

We constructed a 12,008-bp pCps-Tet-mCherry shuttle vector using a fragment of pBOMB4-Tet-mCherry, including genes for the green fluorescent protein (GFP), mCherry, and ampicillin resistance (AmpR) (16), along with the full sequence of the p01DC12 plasmid derived from C. psittaci 01DC12 (Fig. 1A). mCherry can be replaced with various target genes at a multiple cloning site (MCS) using restriction digestion. Target genes can be regulated by a tetracycline-inducible (tet) promoter (Fig. 1B).

The expected plasmid size of 12,008 bp was confirmed by digestion of pCps-Tet-mCherry with NotI (Fig. S2). Sequencing of the pCps-Tet-mCherry plasmid shuttle vector revealed that the full sequence of p01DC12 was 100% identical to the original sequence (GenBank: HF545615.1). The origin of replication in pCps-Tet-mCherry was identical to pGFP::SW2 (12). The amino acid sequence of mCherry was identical to that of the pMCherry-C1 vector (TaKaRa Bio, Saint-Germain-en-Laye, France). Another region of the pBOMB4-Tet-mCherry fragment was 100% identical to the literature data (GenBank: KF790910.1) (16).

10.1128/mSphere.00787-20.2 FIG S2 The digestion of pCps-Tet-mCherry with NotI. Download FIG S2, TIF file, 0.1 MB. Copyright © 2020 Shima et al. 2020 Shima et al. This content is distributed under the terms of the Creative Commons Attribution 4.0 International license.

Since our plasmid shuttle vector was constructed from a plasmid of C. psittaci 01DC12, we selected the same strain as a control to perform our initial transformation. After transformation of C. psittaci 01DC12 with pCps-Tet-mCherry and infection in epithelial cells, a strong GFP signal was detected in C. psittaci 01DC12 (C. psittaci 01DC12-pCps-Tet-mCherry) inclusions at 24 and 48 h postinfection (hpi) (Fig. S3A). Furthermore, mCherry was successfully induced by both 10 and 100 ng/ml anhydrotetracycline hydrochloride (aTC) treatment, and we did not detect mCherry expression in the absence of aTC (Fig. S3A and S3B).

10.1128/mSphere.00787-20.3 FIG S3 GFP expression and mCherry induction in pCps-Tet-mCherry-transformed C. psittaci strain 01DC12. Transformed C. psittaci 01DC12 bacteria were grown in epithelial cells with 1U/ml PEN for 24 and 48 h. (A) GFP fluorescence of chlamydial inclusions was visualized in living cells without fixing and staining. aTC (10 and 100 ng/ml) was added at 1 hpi to induce mCherry expression. Images are representative of three independent experiments. White arrows show chlamydial inclusions. White scale bars represent 10 μm. (B) mCherry was analyzed by Western blotting and densitometric analyses at 24 and 48 hpi. mCherry protein amounts were normalized to chlamydial HSP60. GAPDH was used as a loading control. (B: *n* = 3; mean ± SEM; Sidak’s multiple comparison: ***, *P ≤ *0.001). Download FIG S3, TIF file, 1.5 MB. Copyright © 2020 Shima et al. 2020 Shima et al. This content is distributed under the terms of the Creative Commons Attribution 4.0 International license.

The bovine strain C. psittaci 02DC15 and the psittacine isolate C. psittaci 6BC are categorized in the same *ompA* genotype A. Genetically, C. psittaci 02DC15 belongs to the psittacine C. psittaci 6BC clade (1, 17).

**FIG 2:**
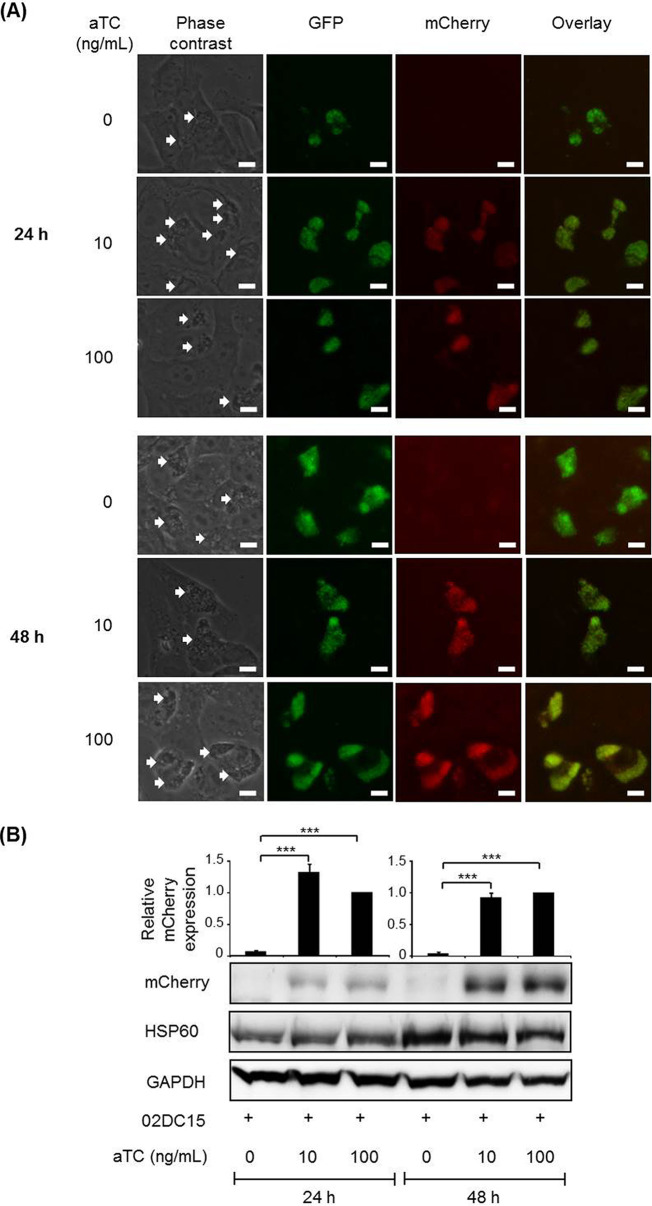
GFP expression and mCherry induction in pCps-Tet-mCherry-transformed C. psittaci strain 02DC15. Transformed C. psittaci 02DC15 bacteria were grown in epithelial cells with 1U/ml PEN for 24 and 48 h. (A) GFP fluorescence of chlamydial inclusions was visualized in living cells without fixing and staining. aTC (10 and 100 ng/ml) was added at 1 hpi to induce mCherry expression. Images are representative of three independent experiments. White arrows show chlamydial inclusions. White scale bars represent 10 μm. (B) mCherry was analyzed by Western blotting and densitometric analyses at 24 and 48 hpi. mCherry protein amounts were normalized to chlamydial HSP60. GAPDH was used as a loading control. (*n* = 4; mean ± SEM; Sidak’s multiple comparison: ***, *P ≤ *0.001).

Due to their genome sequence similarity and biosafety considerations, we next investigated whether C. psittaci strain 02DC15 could be transformed with pCps-Tet-mCherry. Following transformation, we observed not only expression of GFP, but also induction of mCherry in C. psittaci 02DC15 (C. psittaci 02DC15-pCps-Tet-mCherry) inclusions using 10 and 100 ng/ml of aTC (Fig. 2A and B).

### Impact of pCps-Tet-mCherry on the growth of C. psittaci.

**FIG 3:**
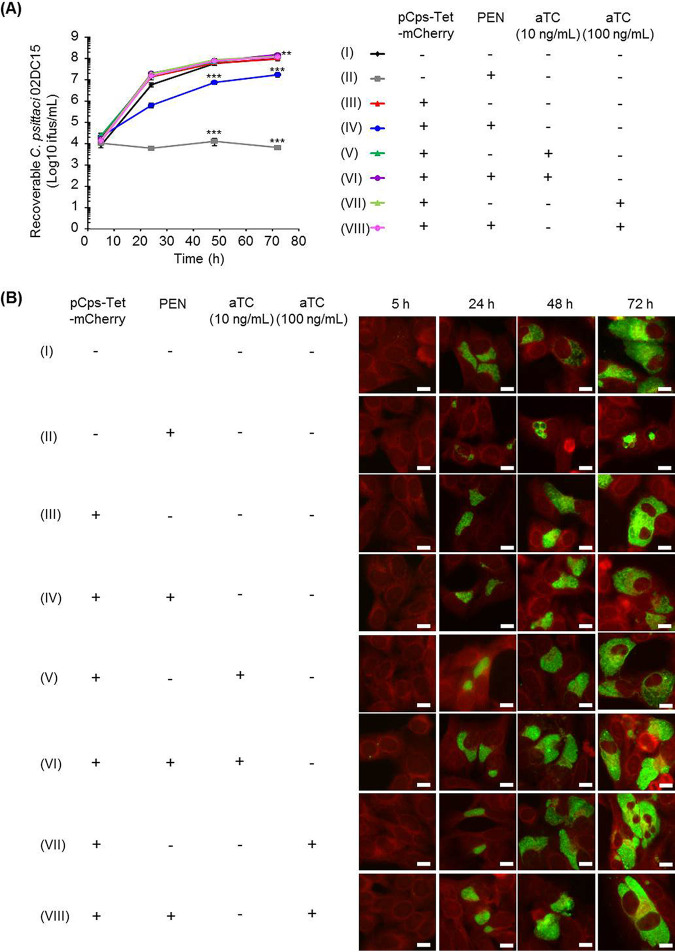
One-step growth curve and inclusion morphology of pCps-Tet-mCherry-transformed and untransformed C. psittaci strain 02DC15. (A) C. psittaci strain 02DC15 transformed or untransformed with pCps-Tet-mCherry was grown in epithelial cells with or without 10 U/ml PEN and 0, 10, or 100 ng/ml of aTC. Recoverable C. psittaci 02DC15 IFUs were determined at 5, 24, 48, and 72 hpi. The numbers of recoverable C. psittaci 02DC15 under each condition (II to VIII) at the indicated time were compared to those of untransformed C. psittaci 02DC15 without PEN (I). (*n* = 3 to 6; mean ± SEM; Sidak’s multiple comparison: **, *P* ≤ 0.01; ***, *P ≤ *0.001). (B) Representative immunofluorescence images of pCps-Tet-mCherry-transformed or untransformed C. psittaci 02DC15 at 5, 24, 48, and 72 hpi. Chlamydial inclusions were stained by FITC-labeled monoclonal chlamydial-LPS antibodies. Evans blue counterstaining of host cells was used for better characterization of intracellular inclusions. Images are representative of 3 to 6 independent experiments. White scale bars represent 10 μm.

We investigated whether the transformation of C. psittaci using pCps-Tet-mCherry affected chlamydial growth and morphological characteristics (Fig. 3 and Fig. S4). While penicillin (PEN) treatment slightly reduced the growth of C. psittaci 02DC15-pCps-Tet-mCherry compared to wild-type C. psittaci 02DC15, C. psittaci 02DC15-pCps-Tet-mCherry showed similar growth characteristics compared to wild-type C. psittaci 02DC15 in the absence of PEN (Fig. 3A). Moreover, we confirmed both 10 and 100 ng/ml of aTC did not inhibit the growth of C. psittaci 02DC15-pCps-Tet-mCherry (Fig. 3A). The same trend was observed using the control strain C. psittaci 01DC12 (Fig. S4A). Immunofluorescence analysis revealed similar inclusion morphology between wild-type and transformed C. psittaci regardless of the presence or absence of aTC (Fig. 3B and Fig. S4B). These data indicate that pCps-Tet-mCherry itself and aTC treatment at concentrations used in this study do not interfere with chlamydial growth characteristics.

10.1128/mSphere.00787-20.4 FIG S4 One-step growth curve and the inclusion morphology of C. psittaci strain 01DC12 transformed with pCps-Tet-mCherry. (A) pCps-Tet-mCherry-transformed and untransformed C. psittaci 01DC12 bacteria were grown in epithelial cells with or without 10 U/ml PEN and 0, 10, or 100 ng/ml of aTC for 5, 24, 48, and 72 hpi. The numbers of recoverable C. psittaci 01DC12 IFUs under each condition (II to VIII) at the indicated time were compared to those of untransformed C. psittaci 01DC12 without PEN (I). (*n* = 3 to 6; mean ± SEM; Sidak’s multiple comparison: *, *P ≤ *0.05; **, *P ≤ *0.01; ***, *P ≤ *0.001). (B) Representative immunofluorescence images of pCps-Tet-mCherry-transformed and untransformed C. psittaci 01DC12 cells at 5, 24, 48, and 72 hpi. Chlamydial inclusions were stained by FITC-labeled monoclonal chlamydial-LPS antibodies. Evans blue counterstaining of host cells was used for better characterization of intracellular inclusions. Images are representative of 3 to 6 independent experiments. White scale bars represent 10 μm. Download FIG S4, TIF file, 2.5 MB. Copyright © 2020 Shima et al. 2020 Shima et al. This content is distributed under the terms of the Creative Commons Attribution 4.0 International license.

### Plasmid retention in transformed C. psittaci.

The average of the endogenous plasmid copy number is 2.0 ± 0.2 per chromosome in the wild-type C. psittaci 02DC15 at 48 hpi (Fig. S5A). In contrast, C. psittaci 02DC15-pCps-Tet-mCherry harbors 3.6 ± 0.1 of the pCps-Tet-mCherry plasmid shuttle vector per chromosome (Fig. S5B). Transformation resulted in 98 ± 0.1% (*n* = 3) reduction of endogenous wild-type plasmid in C. psittaci 02DC15-pCps-Tet-mCherry. A similar trend was observed using the control strain C. psittaci 01DC12 (Fig. S5A and B). While the endogenous plasmid copy number is 1.1 ± 0.2 per chromosome in the strain C. psittaci 01DC12 at 48 hpi (Fig. S5A), C. psittaci 01DC12-pCps-Tet-mCherry harbors 3.0 ± 0.3 pCps-Tet-mCherry plasmid shuttle vector per chromosome (Fig. S5B) and shows a decrease of 96 ± 0.8% of the endogenous plasmid compared to the wild-type strain.

10.1128/mSphere.00787-20.5 FIG S5 Endogenous and pCps-Tet-mCherry plasmid copy numbers. (A) Wild-type C. psittaci 02DC15 and C. psittaci 01DC12 bacteria were cultured in epithelial cells for 48 h. Quantitative PCR was performed using primers specific to genomic DNA or endogenous plasmids. Plasmid copy number was determined relative to genomic DNA. (B) C. psittaci 02DC15-pCps-Tet-mCherry and C. psittaci 01DC12-pCps-Tet-mCherry were cultured in epithelial cells for 48 h. Quantitative PCR was performed using primers specific to genomic DNA or the pCps-Tet-mCherry plasmid. pCps-Tet-mCherry copy number was determined relative to genomic DNA. (*n* = 3; mean ± SEM). Download FIG S5, TIF file, 0.2 MB. Copyright © 2020 Shima et al. 2020 Shima et al. This content is distributed under the terms of the Creative Commons Attribution 4.0 International license.

**FIG 4:**
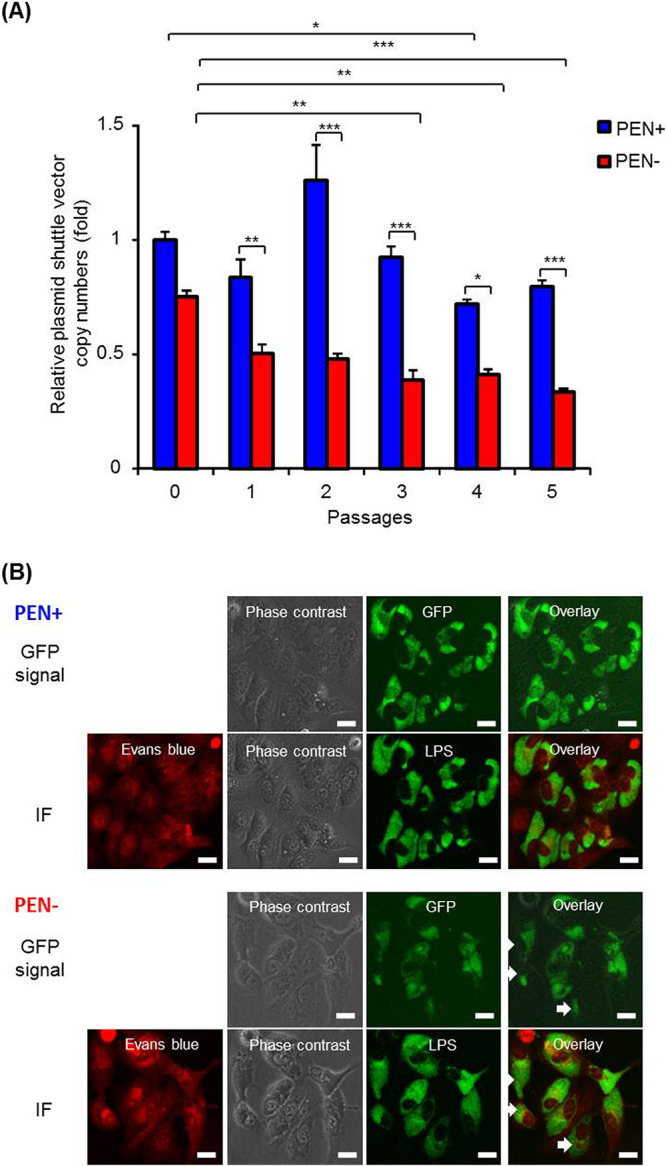
The pCps-Tet-mCherry plasmid can be stably maintained by C. psittaci strain 02DC15 and expresses GFP. (A) pCps-Tet-mCherry-transformed C. psittaci 02DC15 bacteria were subcultured in epithelial cells in the presence or absence of 10 U/ml PEN every 2 days over 5 passages. Quantitative PCR was performed using primers specific to genomic DNA or to the pCps-Tet-mCherry plasmid. Copy numbers of pCps-Tet-mCherry were normalized to genomic DNA at each passage in the presence or absence of PEN, and were compared to passage 0 of C. psittaci 02DC15 in the presence of PEN. (*n* = 3; mean ± SEM, Sidak’s multiple comparison: *, *P ≤ *0.05; **, *P ≤ *0.01; ***, *P ≤ *0.001). (B) Representative GFP and immunofluorescence images of pCps-Tet-mCherry-transformed C. psittaci 02DC15 cells 48 hpi at passage 5. After a GFP signal was detected by fluorescence microscopy, the cells were fixed by methanol. Then, chlamydial inclusions were stained by FITC-labeled monoclonal chlamydial-LPS antibodies. Evans blue counterstaining of host cells was used for better characterization of intracellular inclusions. IF, immunofluorescence; LPS, lipopolysaccharide. White arrows show C. psittaci 02DC15 that lost pCps-Tet-mCherry during passages. White scale bars represent 20 μm.

Plasmids are generally prone to being lost without selection pressure. We next investigated the shuttle vector/plasmid stability in transformed C. psittaci. Plasmid shuttle vector pCps-Tet-mCherry was stably maintained in both C. psittaci 02DC15-pCps-Tet-mCherry and the control strain C. psittaci 01DC12-pCps-Tet-mCherry in the presence of PEN (Fig. 4A and Fig. S6A). Although copy numbers were similar in the presence or absence of PEN at initial culture, pCps-Tet-mCherry was significantly reduced in the absence of PEN from passage 1 (Fig. 4A and B, Fig. S6A and B). This indicates that the pCps-Tet-mCherry plasmid could be stably retained with selection pressure for long-term maintenance.

10.1128/mSphere.00787-20.6 FIG S6 The pCps-Tet-mCherry plasmid can be stably retained in C. psittaci 01DC12 and expresses GFP. (A) pCps-Tet-mCherry-transformed C. psittaci 01DC12 bacteria were subcultured in epithelial cells in the presence or absence of 10 U/ml PEN every 2 days over 5 passages. Quantitative PCR was performed using primers specific to genomic DNA or the pCps-Tet-mCherry plasmid. Copy numbers of pCps-Tet-mCherry were normalized to genomic DNA at each passage in the presence or absence of PEN and were compared to passage 0 of C. psittaci 01DC12 in the presence of PEN (*n* = 3; mean ± SEM; Sidak’s multiple comparison: *, *P ≤ *0.05; **, *P ≤ *0.01; ***, *P ≤ *0.001). (B) Representative GFP and immunofluorescence images of pCps-Tet-mCherry-transformed C. psittaci 01DC12 cells at 48 hpi at passage 5. After a GFP signal was detected by fluorescence microscopy, the cells were fixed by methanol. Then, chlamydial inclusions were stained by FITC-labeled monoclonal chlamydial-LPS antibodies. Evans blue counterstaining of host cells was used for better characterization of intracellular inclusions. IF, immunofluorescence; LPS, lipopolysaccharide. White arrows show C. psittaci 01DC12 that lost pCps-Tet-mCherry during passages. White scale bars represent 20 μm. Download FIG S6, TIF file, 1.6 MB. Copyright © 2020 Shima et al. 2020 Shima et al. This content is distributed under the terms of the Creative Commons Attribution 4.0 International license.

### Impact of aTC on host cell metabolism.

It is known that a long-term treatment of tetracycline inhibits host cell metabolism, especially mitochondrial function (18). Therefore, we investigated the impact of aTC on mitochondrial activity.

**FIG 5:**
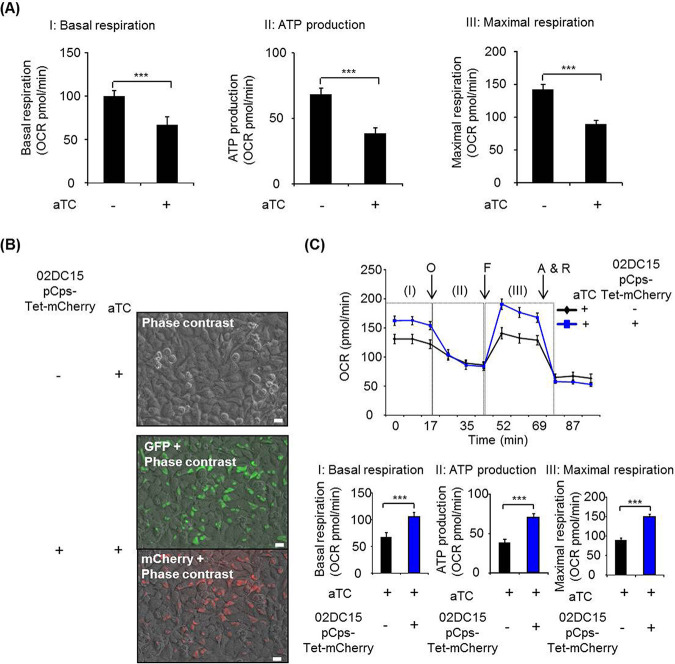
Analysis of mitochondrial activity in cells treated with aTC and infected with C. psittaci 02DC15-pCps-Tet-mCherry. (A) The effect of aTC (10 ng/ml) on mitochondrial activity in noninfected epithelial cells. Mitochondrial activity shown by oxygen consumption rate (OCR) was measured by a Mito Stress test kit at 24 hpi. I, II, and III indicate basal respiration, ATP production, and maximal respiration, respectively. (B) Phase contrast image of control cells and image overlay of phase contrast and GFP or mCherry in pCps-Tet-mCherry-transformed C. psittaci 02DC15-infected cells with 10 ng/ml of aTC. White scale bars represent 20 μm. (C) Mitochondrial activity in control cells and pCps-Tet-mCherry-transformed C. psittaci 02DC15-infected cells with 10 ng/ml aTC at 24 hpi. Arrows show sequential injection of different chemical compounds: O, oligomycin; F, FCCP; A & R, antimycin A plus rotenone. I, II, and III indicate basal respiration, ATP production, and maximal respiration, respectively. Images are representative of three independent experiments. (A: *n* = 13 from five independent experiments, C: *n* = 13 from five independent experiments; mean ± SEM, Student’s *t* test: ***, *P ≤ *0.001).

aTC (10 ng/ml) at the concentration used in this study attenuated host cell mitochondrial activity, as indicated by basal respiration, ATP production, and maximal respiration (Fig. 5A). This indicates that we have to take into account the effect of aTC on host cell functions. However, when we compared mitochondrial activity in aTC-treated uninfected control cells to aTC-treated C. psittaci 02DC15-pCps-Tet-mCherry infected cells, significantly upregulated mitochondrial activity was observed in aTC-treated C. psittaci 02DC15-pCps-Tet-mCherry infected cells (Fig. 5B and C). This upregulation was similar to that observed in wild-type C. psittaci 02DC15 inclusion-bound mitochondria (Fig. S7A and B).

10.1128/mSphere.00787-20.7 FIG S7 Analysis of mitochondrial activity during wild-type C. psittaci 02DC15 infection. (A) Transmission electron microscope (TEM) analysis of mitochondrial interactions with wild-type C. psittaci 02DC15 at 24 hpi. Black scale bars represent 2 μm. Images are representative of three independent experiments. Yellow arrow head, mitochondrion. (B) Analysis of mitochondrial activity during wild-type C. psittaci 02DC15 infection. Mitochondrial activity shown by OCR was measured by Mito Stress test kit at 24 hpi. Arrows show sequential injection of different chemical compounds: O, oligomycin; F, FCCP; A & R, antimycin A plus rotenone. I, II, and III indicate basal respiration, ATP production, and maximal respiration, respectively. (*n* = 21 from eight independent experiments; mean ± SEM, Student’s *t* test: *, *P ≤ *0.05). Download FIG S7, TIF file, 1.2 MB. Copyright © 2020 Shima et al. 2020 Shima et al. This content is distributed under the terms of the Creative Commons Attribution 4.0 International license.

## DISCUSSION

C. psittaci has the capability of causing severe disease in animals and humans (1–3). However, due to the lack of genetic manipulation tools for this important pathogen, molecular studies of C. psittaci are lagging far behind that of other human chlamydial pathogens, such as C. trachomatis.

Development of chlamydial shuttle vectors and stable transformation of C. trachomatis, C. muridarum, and C. pneumoniae have been established only in the last decade (12–14, 19). Bauler and Hackstadt further developed a novel plasmid shuttle vector pBOMB4-Tet-mCherry, which encodes the Tet-inducible promoter system for the expression of recombinant target proteins in C. trachomatis (16). Moreover, Weber et al. were able to identify 10 novel inclusion membrane proteins in C. trachomatis infection using this shuttle vector system (20).

Considering there are some barriers to plasmid replication (13–15) and the advantages of pBOMB4-Tet-mCherry reporter system, the C. psittaci backbone plasmid shuttle vector pCps-Tet-mCherry was constructed from the full sequence of p01DC12 and the fragment of C. trachomatis shuttle vector pBOMB4-Tet-mCherry. We showed that GFP was expressed after transformation of the control strain C. psittaci 01DC12 using pCps-Tet-mCherry. Since avian C. psittaci strains cause severe lung diseases in humans, it is important to assess whether pCps-Tet-mCherry system works in avian C. psittaci strains, including C. psittaci 6BC. However, conducting the experiment using C. psittaci 6BC is generally troublesome due to stricter regulations for the biosafety level. Therefore, we used C. psittaci 02DC15 as a substitute for avian C. psittaci 6BC. While whole-genome analysis using UpSetR (21) revealed a total of 984 CDS in C. psittaci 6BC and 991 CDS in C. psittaci 02DC15 genomes (Fig. S8), we were able to show that all of the genes present in C. psittaci 6BC are encountered in C. psittaci 02DC15 as well, thus indicating their genetic equivalence. Due to the genome similarity between C. psittaci 6BC and C. psittaci 02DC15, C. psittaci 02DC15 is widely used for a number of immunological and cell biological studies, as well as animal infection trials (22–28). Reinhold et al. established a bovine respiratory model of acute C. psittaci infection using strain 02DC15 (27). Koch-Edelmann et al. demonstrated the importance of lipid metabolism in C. psittaci 02DC15 infection (25). Dutow et al. elucidated the role of effector of the complement (C3a) in C. psittaci 02DC15 infection (22). Most recently, Radomski et al. reported that C. psittaci 02DC15 infection of dendritic cells enhances exosome release, resulting in strong induction of IFN-γ production by natural killer cells and enhancement of apoptosis (24). Therefore, successful transformation using C. psittaci 02DC15 is particularly meaningful with respect to the biology of C. psittaci infection.

10.1128/mSphere.00787-20.8 FIG S8 Whole-genome comparison of C. psittaci 02DC15 and C. psittaci 6BC with type strains of 11 other species of *Chlamydia*. The analysis was performed using UpSetR. C. psittaci 02DC15 and C. psittaci 6BC as well as C. caviae (a close relative of C. psittaci) (39) are highlighted in red. While 790 CDSs are shared by all 13 chlamydial strains, a total of 991 and 984 CDSs were identified in the genomes of C. psittaci 02DC15 and C. psittaci 6BC, respectively, (at 95% similarity threshold). Counting of all individual bars also reveals that all of the genes present in C. psittaci 6BC are encountered in C. psittaci 02DC15. Cmu, Chlamydia muridarum; Csu, Chlamydia suis; Ctr_A, Chlamydia trachomatis serovar A; Cga, Chlamydia gallinacea; Cpe, Chlamydia pecorum; Cav, Chlamydia avium; Cib, Chlamydia ibidis; Cfe, Chlamydia felis; Cps, Chlamydia psittaci; Cca, Chlamydia caviae; Cab, Chlamydia abortus; Cpn, Chlamydia pneumoniae. Download FIG S8, PDF file, 0.1 MB. Copyright © 2020 Shima et al. 2020 Shima et al. This content is distributed under the terms of the Creative Commons Attribution 4.0 International license.

In addition to transformation of C. psittaci 02DC15, the induction of mCherry expression was controlled by aTC treatment. In this study, mCherry was used as an exogenous gene, but it is possible to insert other target genes into the MCS. Therefore, the pCps-Tet-mCherry is a useful plasmid shuttle vector to investigate the function of specific proteins during C. psittaci infection.

A tetracycline-inducible system is beneficial for biological research. However, it has been demonstrated that >100 ng/ml of tetracycline or its derivatives attenuates mitochondrial activity (18, 29, 30). Since mitochondrial function is essential for chlamydiae to sustain their life (8, 31, 32), we investigated the impact of aTC on mitochondria in C. psittaci infection.

Oxygen consumption of C. psittaci 02DC15-pCps-Tet-mCherry-infected cells was measured by a Seahorse XF analyzer with solid state sensor probes containing polymer embedded fluorophores (33). The peak absorption and emission of the oxygen sensor are 530 and 650 nm, respectively. Although C. psittaci 02DC15-pCps-Tet-mCherry expresses GFP and mCherry, intracellular dye fluorescence does not interfere with optical sensors given enough distance to the object, as various groups have demonstrated (34, 35).

Similar to previous studies (18, 29, 30), 10 ng/ml of aTC attenuated the mitochondrial oxygen consumption rate in noninfected epithelial cells. However, we could show that mitochondrial activity was significantly upregulated in C. psittaci 02DC15-pCps-Tet-mCherry-infected cells under aTC treatment, similar to that observed in wild-type C. psittaci 02DC15 infection in the absence of aTC. These data indicate that pCps-Tet-mCherry can be a beneficial tool for further biological studies in C. psittaci infection, though we need to take into account the impact of aTC on host cell functions.

Recently, gene knockout techniques such as the TargeTron system have also been established in C. trachomatis (36, 37). Our plasmid shuttle vector pCps-Tet-mCherry and the future development of a knockout system are promising new approaches to elucidate chlamydial protein functions during C. psittaci infection.

Taken together, our findings highlight that pCps-Tet-mCherry can be used for C. psittaci transformation and functional studies. This system will enable the identification of novel virulence factors and improvement of treatment strategies for C. psittaci infection.

## MATERIALS AND METHODS

### Bacterial strains, epithelial cells, chemicals, and antibodies.

Animal-derived isolates C. psittaci 01DC12 (*ompA* genotype E, pigeon clade) and C. psittaci 02DC15 (*ompA* genotype A, psittacine clade) were provided by the National and OIE Reference Laboratory for Chlamydioses at FLI Jena (1). HeLa cells (ATCC CCL-2)/HEp-2 cells (ATCC CCL-23) were used for the growth of C. psittaci. All chemicals were purchased from Sigma-Aldrich (Deisenhofen, Germany). Rabbit anti-mCherry and mouse anti-heat shock protein 60 (HSP60) were purchased from Thermo Fisher Scientific (Waltham, MA). Rabbit anti-glyceraldehyde-3-phosphate dehydrogenase (GAPDH), horseradish peroxidase (HRP)-horse anti-mouse IgG and HRP-goat anti-rabbit IgG were purchased from Cell Signaling Technology (Frankfurt am Main, Germany).

### Cell culture medium.

Dulbecco modified Eagle medium (DMEM) was supplemented with 10% fetal bovine serum (FBS) (Invitrogen), 1 mM sodium pyruvate (Pan-Biotech GmbH, Aidenbach, Germany) and 30 mM HEPES. RPMI 1640 medium was supplemented with 5% FBS (Invitrogen), nonessential amino acids (Gibco/Thermo Fisher Scientific), and 2 mM l-glutamine (Lonza, Walkersville, MD).

### Construction of C. psittaci-derived plasmid shuttle vector.

The plasmid vector pCps-Tet-mCherry was constructed using NEBuilder HiFi DNA assembly cloning kit (New England BioLabs [NEB], Ipswich, MA). Using Phusion Hot Start II High-Fidelity DNA polymerase (Thermo Fisher Scientific), the full sequence of p01DC12 was amplified from DNA of C. psittaci 01DC12 (7,553 bp, GenBank: HF545615.1). The fragment, including the tet promoter, mCherry, MCS (NotI, PstI, KpnI and SalI), AmpR, pUC origin of replication (ori), the meningococcal class I protein promoter (MCIP) derived from Neisseria meningitidis MC50, and GFP, was amplified from pBOMB4-Tet-mCherry (16). The plasmid vector pBOMB4-Tet-mCherry was kindly provided by Ted Hackstadt (National Institutes of Allergy and Infectious Disease, NIH, Hamilton, MT, USA). These two segments were assembled following the NEB manufacturer’s instructions, resulting in pCps-Tet-mCherry. Primer sets are listed in Table S1.

10.1128/mSphere.00787-20.9 TABLE S1 Primers used for PCR and sequencing. Download Table S1, XLSX file, 0.01 MB. Copyright © 2020 Shima et al. 2020 Shima et al. This content is distributed under the terms of the Creative Commons Attribution 4.0 International license.

### Genetic transformation.

Transformation was performed as described in a previous study (13). pCps-Tet-mCherry was extracted from a Dam- and Dcm-methylase-deficient strain, E. coli GM2163, using a Qiagen plasmid mega kit (Hilden, Germany) (12). PEN (1U/ml) was used for the selection of transformed C. psittaci. A mixture of C. psittaci 01DC12 or 02DC15 1 × 10^8^ inclusion forming units (IFU) and 15 μg of pCps-Tet-mCherry were incubated in 200 μl calcium chloride buffer (10 mM Tris, 50 mM calcium chloride, pH 7.4) for 30 min at room temperature. Then, 200 μl of C. psittaci mixed with pCps-Tet-mCherry were incubated with 200 μl of trypsinized 8 × 10^6^ epithelial cells in calcium chloride buffer for 20 min at room temperature with mild agitation.

The total volume (400 μl) of the mixture of epithelial cells, pCps-Tet-mCherry, and C. psittaci 01DC12 or 02DC15 was distributed over 4 wells (each 100 μl) containing 2 ml DMEM medium and 1 μg/ml cycloheximide in a 6-well plate and incubated at 37°C under 5% CO_2_. At 24 hpi, 1U/ml PEN was added to each well and further incubated for 48 h at 37°C under 5% CO_2_. Afterward, infected epithelial cells were scraped and lysed with glass beads.

Freshly prepared epithelial cells were infected with transformed C. psittaci in 6-well plates containing 5 ml DMEM medium with 1 μg/ml cycloheximide and 1U/ml PEN. Passages were performed every 2 to 3 days. GFP expression was observed by passage 2.

### Induction of mCherry.

A total of 2 × 10^5^ epithelial cells in 24-well plates were infected with 2 × 10^5^ IFU of transformed C. psittaci in DMEM medium. At 1 hpi, 10 or 100 ng/ml of aTC was added to induce mCherry.

### Western blot analysis.

To determine the amount of mCherry protein, 1 × 10^6^ cells were seeded in 6-well plates (Greiner bio-one, Frickenhausen, Germany) and infected with C. psittaci 02DC15-pCps-Tet-mCherry or C. psittaci 01DC12-pCps-Tet-mCherry (1 IFUs/cell). At 1 hpi, 10 or 100 ng/ml of aTC was added to induce mCherry. C. psittaci-infected cells were lysed by 8 M urea supplemented with 325 U/ml Benzonase nuclease (Sigma-Aldrich) at the indicated times. Cell lysates were diluted into Laemmlie buffer (50 mM Tris-HCl pH 6.8, 2% SDS, 1% 2-mercaptoethanol, 10% glycerol, 0.1% bromophenol blue). Samples were analyzed by Western blot analysis (Bio-Rad, Hercules, CA) and visualized using enhanced chemiluminescence (ECL) reagent (Millipore and Thermo Fisher Scientific). Images were acquired by Fusion FX7 (Vilber Lourmat, Eberhardzell, Germany) and the density of each band was measured by Bio-1D software (Vilber Lourmat).

### Recovery assay.

A total of 5 × 10^4^ epithelial cells in 1 ml DMEM medium were seeded into 24-well plates (Greiner bio-one) and cultured overnight at 37˚C under 5% CO_2_. For the infection, 2 × 10^5^ IFUs of either untransformed or pCps-Tet-mCherry-transformed C. psittaci strains as well as cycloheximide (1 μg/ml) were added to each well. When appropriate, PEN (10 U/ml) and 0, 10, or 100 ng/ml of aTC were added at 1 hpi. The plate was centrifuged at 700 × *g* for 1 h at 35˚C and incubated for 5, 24, 48, and 72 h. After the indicated time, the cells were subsequently cultured for 48 h for determination of the recoverable C. psittaci.

### Real-time quantitative PCR.

Determination of plasmid copy number was performed as demonstrated previously (16). Wild type or pCps-Tet-mCherry-transformed C. psittaci were cultured in epithelial cells in the presence or absence of 10 U/ml PEN. Afterward, isolated C. psittaci were boiled in 20 mM dithiothreitol (DTT) for 15 min and the supernatant was collected to obtain the genomic and plasmid DNA mixture. One primer set was designed to detect the *hctA* gene (GenBank, AEG87858.1; CPS0B_0937) in the genome. Another primer set was designed to detect the GFP gene for quantitation of the pCps-Tet-mCherry plasmid shuttle vector. For the endogenous plasmid, primers were designed to detect CDS5 or the CDS1-CDS2 junction, which is separated by the fragment of pBOMB4-Tet-mCherry. Real-time quantitative PCR was performed using LightCycler 480 SYBR green I Master on LightCycler 480 II (Roche Molecular Biochemicals, Mannheim, Germany). Plasmid copy number was calculated using the threshold cycle (2^ΔΔ^*^CT^*) method (38) relative to genomic DNA.

### Plasmid stability.

A total of 3 × 10^5^ epithelial cells in 1 ml DMEM medium were initially infected with pCps-Tet-mCherry-transformed C. psittaci at 0.5 IFUs/cell in the presence or absence of 10 U/ml PEN. The infected cells were subcultured every 2 days for 5 times. From the second passage, epithelial cells were infected with serial dilutions of C. psittaci at 0.5 to 0.8 IFUs/cell. To determine plasmid copy number, extracted genomic and plasmid DNA mixture was analyzed as described above. At passage 5, the GFP signal was detected by fluorescence microscopy and cells were fixed by methanol. Afterward, chlamydial inclusions were stained by fluorescein isothiocyanate (FITC)-labeled monoclonal chlamydial lipopolysaccharide (LPS) antibodies.

### Fluorescence microscopy.

A fluorescence microscope Keyence BZ-9000 (Keyence, Osaka, Japan) was used to detect the fluorescence signal of GFP or mCherry expressed in C. psittaci. In addition, untransformed or pCps-Tet-mCherry-transformed C. psittaci-infected cells were analyzed by immunofluorescence staining with mouse anti-chlamydial LPS antibody (green), which stained chlamydial inclusions. Evans blue was used for counterstaining of host cells (red).

### Transmission electron microscopy.

C. psittaci 02DC15-infected cells were fixed with 2% paraformaldehyde and 2.5% glutaraldehyde in 0.1 M cacodylate buffer for 1 h. Postfixation was performed with 1% OsO_4_ in 0.1 M cacodylate buffer for 2 h. Samples were dehydrated with a graded ethanol series and embedded in araldite (Fluka, Buchs, Switzerland). Ultrathin sections were stained with uranyl acetate and lead citrate and were examined with a JEOL 1011 transmission electron microscope (TEM) (JEOL, Tokyo, Japan).

### Plasmid sequencing.

Extracted pCps-Tet-mCherry plasmid was sequenced at Eurofins Genomics (Ebersberg, Germany). Primer sets are listed in Table S1.

### Plasmid maps.

Plasmid maps were made by SnapGene. Using CLUSTALW in GenomeNet (http://www.genome.jp/), p01DC12 plasmid sequences were compared to pCps6BC.

### Genome comparion of C. psittaci 02DC15 and C. psittaci 6BC.

UpSetR package was used in the genome comparison (21). A total of 13 genomes (12 species of *Chlamydia*) and their homologous gene groups identified with genome analysis pipeline RIBAP were selected as input for UpSetR.

### Metabolic analysis.

A total of 2 × 10^4^ HeLa cells in RPMI 1640 were treated with 10 ng/ml aTC. In addition, cells were infected with untransformed or pCps-Tet-mCherry-transformed C. psittaci without cycloheximide in 24-well XF plates (Agilent, Santa Clara, CA). Plates were centrifuged at 700 × *g* for 1 h at 35˚C and incubated for 24 h. Afterward, Mito Stress test kits were used following Seahorse Bioscience manufacturer’s instructions with chemical concentrations as follows: oligomycin (0.5 μM), FCCP (0.2 μM), and antimycin A (1 μM) plus rotenone (1 μM). Before the assay of pCps-Tet-mCherry transformed-C. psittaci infected cells, expression of GFP and mCherry was detected by fluorescence microscopy BZ-9000 (Keyence).

### Statistics.

Data are indicated as means ± standard error of the mean (SEM). Statistical analyses were performed by GraphPad Prism 7 statistical software. When three or more groups were compared in the experiment, Sidak’s multiple comparison was used in cases where one-way analysis of variance showed statistical significance (*P* values ≤0.05). Data between two groups were evaluated using Student’s *t* test. In Sidak’s multiple comparison and Student’s *t* test, *P* values of ≤0.05 were considered statistically significant.

### Data availability.

The sequence of the pCps-Tet-mCherry plasmid shuttle vector reported is available in the DDBJ/EMBL/GenBank databases under accession number LC548057.

## References

[1] Knittler MR, Sachse K. 2015. Chlamydia psittaci: update on an underestimated zoonotic agent. Pathog Dis 73:1–15. doi:10.1093/femspd/ftu007. 25853998

[2] Beeckman DS, Vanrompay DC. 2009. Zoonotic Chlamydophila psittaci infections from a clinical perspective. Clin Microbiol Infect 15:11–17. doi:10.1111/j.1469-0691.2008.02669.x. 19220335

[3] Sachse K, Laroucau K, Vanrompay D. 2015. Avian chlamydiosis. Curr Clin Micro Rpt 2:10–21. doi:10.1007/s40588-014-0010-y.

[4] Campbell LA, Kuo CC. 2004. Chlamydia pneumoniae—an infectious risk factor for atherosclerosis? Nat Rev Microbiol 2:23–32. doi:10.1038/nrmicro796. 15035006

[5] Stephens RS, Kalman S, Lammel C, Fan J, Marathe R, Aravind L, Mitchell W, Olinger L, Tatusov RL, Zhao Q, Koonin EV, Davis RW. 1998. Genome sequence of an obligate intracellular pathogen of humans: Chlamydia trachomatis. Science 282:754–759. doi:10.1126/science.282.5389.754. 9784136

[6] Kading N, Szaszak M, Rupp J. 2014. Imaging of Chlamydia and host cell metabolism. Future Microbiol 9:509–521. doi:10.2217/fmb.14.13. 24810350

[7] Subtil A. 2011. Rerouting of host lipids by bacteria: are you CERTain you need a vesicle? PLoS Pathog 7:e1002208. doi:10.1371/journal.ppat.1002208. 21909265 PMC3164641

[8] Voigt A, Schofl G, Saluz HP. 2012. The Chlamydia psittaci genome: a comparative analysis of intracellular pathogens. PLoS One 7:e35097. doi:10.1371/journal.pone.0035097. 22506068 PMC3323650

[9] Schofl G, Voigt A, Litsche K, Sachse K, Saluz HP. 2011. Complete genome sequences of four mammalian isolates of Chlamydophila psittaci. J Bacteriol 193:4258. doi:10.1128/JB.05382-11. 21705611 PMC3147682

[10] Voigt A, Schofl G, Heidrich A, Sachse K, Saluz HP. 2011. Full-length de novo sequence of the Chlamydophila psittaci type strain, 6BC. J Bacteriol 193:2662–2663. doi:10.1128/JB.00236-11. 21441521 PMC3133153

[11] Binet R, Maurelli AT. 2009. Transformation and isolation of allelic exchange mutants of Chlamydia psittaci using recombinant DNA introduced by electroporation. Proc Natl Acad Sci U S A 106:292–297. doi:10.1073/pnas.0806768106. 19104068 PMC2629194

[12] Wang Y, Kahane S, Cutcliffe LT, Skilton RJ, Lambden PR, Clarke IN. 2011. Development of a transformation system for Chlamydia trachomatis: restoration of glycogen biosynthesis by acquisition of a plasmid shuttle vector. PLoS Pathog 7:e1002258. doi:10.1371/journal.ppat.1002258. 21966270 PMC3178582

[13] Shima K, Wanker M, Skilton RJ, Cutcliffe LT, Schnee C, Kohl TA, Niemann S, Geijo J, Klinger M, Timms P, Rattei T, Sachse K, Clarke IN, Rupp J. 2018. The genetic transformation of Chlamydia pneumoniae. mSphere 3:e00412-18. doi:10.1128/mSphere.00412-18. 30305318 PMC6180227

[14] Wang Y, Cutcliffe LT, Skilton RJ, Ramsey KH, Thomson NR, Clarke IN. 2014. The genetic basis of plasmid tropism between Chlamydia trachomatis and Chlamydia muridarum. Pathog Dis 72:19–23. doi:10.1111/2049-632X.12175. 24700815 PMC4314687

[15] Song L, Carlson JH, Zhou B, Virtaneva K, Whitmire WM, Sturdevant GL, Porcella SF, McClarty G, Caldwell HD. 2014. Plasmid-mediated transformation tropism of chlamydial biovars. Pathog Dis 70:189–193. doi:10.1111/2049-632X.12104. 24214488 PMC3959247

[16] Bauler LD, Hackstadt T. 2014. Expression and targeting of secreted proteins from Chlamydia trachomatis. J Bacteriol 196:1325–1334. doi:10.1128/JB.01290-13. 24443531 PMC3993338

[17] Read TD, Joseph SJ, Didelot X, Liang B, Patel L, Dean D. 2013. Comparative analysis of Chlamydia psittaci genomes reveals the recent emergence of a pathogenic lineage with a broad host range. mBio 4:e00604-12. doi:10.1128/mBio.00604-12. PMC3622922 23532978

[18] Ahler E, Sullivan WJ, Cass A, Braas D, York AG, Bensinger SJ, Graeber TG, Christofk HR. 2013. Doxycycline alters metabolism and proliferation of human cell lines. PLoS One 8:e64561. doi:10.1371/journal.pone.0064561. 23741339 PMC3669316

[19] Agaisse H, Derre I. 2013. A C. trachomatis cloning vector and the generation of C. trachomatis strains expressing fluorescent proteins under the control of a C. trachomatis promoter. PLoS One 8:e57090. doi:10.1371/journal.pone.0057090. 23441233 PMC3575495

[20] Weber MM, Bauler LD, Lam J, Hackstadt T. 2015. Expression and localization of predicted inclusion membrane proteins in Chlamydia trachomatis. Infect Immun 83:4710–4718. doi:10.1128/IAI.01075-15. 26416906 PMC4645406

[21] Conway JR, Lex A, Gehlenborg N. 2017. UpSetR: an R package for the visualization of intersecting sets and their properties. Bioinformatics 33:2938–2940. doi:10.1093/bioinformatics/btx364. 28645171 PMC5870712

[22] Dutow P, Fehlhaber B, Bode J, Laudeley R, Rheinheimer C, Glage S, Wetsel RA, Pabst O, Klos A. 2014. The complement C3a receptor is critical in defense against Chlamydia psittaci in mouse lung infection and required for antibody and optimal T cell response. J Infect Dis 209:1269–1278. doi:10.1093/infdis/jit640. 24273177 PMC3969542

[23] Radomski N, Franzke K, Matthiesen S, Karger A, Knittler MR. 2019. NK cell-mediated processing of Chlamydia psittaci drives potent anti-bacterial Th1 immunity. Sci Rep 9:4799. doi:10.1038/s41598-019-41264-4. 30886314 PMC6423132

[24] Radomski N, Karger A, Franzke K, Liebler-Tenorio E, Jahnke R, Matthiesen S, Knittler MR. 2019. Chlamydia psittaci-infected dendritic cells communicate with NK cells via exosomes to activate antibacterial immunity. Infect Immun 88:e00541-19. doi:10.1128/IAI.00541-19. 31658957 PMC6921653

[25] Koch-Edelmann S, Banhart S, Saied EM, Rose L, Aeberhard L, Laue M, Doellinger J, Arenz C, Heuer D. 2017. The cellular ceramide transport protein CERT promotes Chlamydia psittaci infection and controls bacterial sphingolipid uptake. Cell Microbiol 19. doi:10.1111/cmi.12752. 28544656

[26] Fiegl D, Kagebein D, Liebler-Tenorio EM, Weisser T, Sens M, Gutjahr M, Knittler MR. 2013. Amphisomal route of MHC class I cross-presentation in bacteria-infected dendritic cells. J Immunol 190:2791–2806. doi:10.4049/jimmunol.1202741. 23418629

[27] Reinhold P, Ostermann C, Liebler-Tenorio E, Berndt A, Vogel A, Lambertz J, Rothe M, Ruttger A, Schubert E, Sachse K. 2012. A bovine model of respiratory Chlamydia psittaci infection: challenge dose titration. PLoS One 7:e30125. doi:10.1371/journal.pone.0030125. 22299031 PMC3267716

[28] Ostermann C, Ruttger A, Schubert E, Schrodl W, Sachse K, Reinhold P. 2013. Infection, disease, and transmission dynamics in calves after experimental and natural challenge with a Bovine Chlamydia psittaci isolate. PLoS One 8:e64066. doi:10.1371/journal.pone.0064066. 23691148 PMC3653844

[29] Duewelhenke N, Krut O, Eysel P. 2007. Influence on mitochondria and cytotoxicity of different antibiotics administered in high concentrations on primary human osteoblasts and cell lines. Antimicrob Agents Chemother 51:54–63. doi:10.1128/AAC.00729-05. 17088489 PMC1797653

[30] Riesbeck K, Bredberg A, Forsgren A. 1990. Ciprofloxacin does not inhibit mitochondrial functions but other antibiotics do. Antimicrob Agents Chemother 34:167–169. doi:10.1128/aac.34.1.167. 2327755 PMC171543

[31] Kubo A, Stephens RS. 2001. Substrate-specific diffusion of select dicarboxylates through Chlamydia trachomatis PorB. Microbiology 147:3135–3140. doi:10.1099/00221287-147-11-3135. 11700364

[32] Chowdhury SR, Reimer A, Sharan M, Kozjak-Pavlovic V, Eulalio A, Prusty BK, Fraunholz M, Karunakaran K, Rudel T. 2017. Chlamydia preserves the mitochondrial network necessary for replication via microRNA-dependent inhibition of fission. J Cell Biol 216:1071–1089. doi:10.1083/jcb.201608063. 28330939 PMC5379946

[33] Wu M, Neilson A, Swift AL, Moran R, Tamagnine J, Parslow D, Armistead S, Lemire K, Orrell J, Teich J, Chomicz S, Ferrick DA. 2007. Multiparameter metabolic analysis reveals a close link between attenuated mitochondrial bioenergetic function and enhanced glycolysis dependency in human tumor cells. Am J Physiol Cell Physiol 292:C125–C136. doi:10.1152/ajpcell.00247.2006. 16971499

[34] Kooragayala K, Gotoh N, Cogliati T, Nellissery J, Kaden TR, French S, Balaban R, Li W, Covian R, Swaroop A. 2015. Quantification of oxygen consumption in retina ex vivo demonstrates limited reserve capacity of photoreceptor mitochondria. Invest Ophthalmol Vis Sci 56:8428–8436. doi:10.1167/iovs.15-17901. 26747773 PMC4699410

[35] Okkelman IA, Neto N, Papkovsky DB, Monaghan MG, Dmitriev RI. 2020. A deeper understanding of intestinal organoid metabolism revealed by combining fluorescence lifetime imaging microscopy (FLIM) and extracellular flux analyses. Redox Biol 30:101420. doi:10.1016/j.redox.2019.101420. 31935648 PMC6957829

[36] Weber MM, Noriea NF, Bauler LD, Lam JL, Sager J, Wesolowski J, Paumet F, Hackstadt T. 2016. A functional core of IncA is required for Chlamydia trachomatis inclusion fusion. J Bacteriol 198:1347–1355. doi:10.1128/JB.00933-15. 26883826 PMC4859576

[37] Weber MM, Lam JL, Dooley CA, Noriea NF, Hansen BT, Hoyt FH, Carmody AB, Sturdevant GL, Hackstadt T. 2017. Absence of specific Chlamydia trachomatis inclusion membrane proteins triggers premature inclusion membrane lysis and host cell death. Cell Rep 19:1406–1417. doi:10.1016/j.celrep.2017.04.058. 28514660 PMC5499683

[38] Livak KJ, Schmittgen TD. 2001. Analysis of relative gene expression data using real-time quantitative PCR and the 2(-Delta Delta C(T)) Method. Methods 25:402–408. doi:10.1006/meth.2001.1262. 11846609

[39] Everett KD, Bush RM, Andersen AA. 1999. Emended description of the order Chlamydiales, proposal of Parachlamydiaceae fam. nov. and Simkaniaceae fam. nov., each containing one monotypic genus, revised taxonomy of the family Chlamydiaceae, including a new genus and five new species, and standards for the identification of organisms. Int J Syst Bacteriol 49 Pt 2:415–440. doi:10.1099/00207713-49-2-415. 10319462

